# Insidious-Onset Indurated Plaques on the Shins

**DOI:** 10.3390/dermatopathology8020024

**Published:** 2021-06-06

**Authors:** Rishi Agrawal, Daniel Knabel, Anthony P. Fernandez

**Affiliations:** 1 Imperial College School of Medicine, Imperial College London, South Kensington, London SW7 2BU, UK; 2 Departments of Dermatology, Cleveland Clinic, Cleveland, OH 44195, USA; drknabel@gmail.com (D.K.); fernana6@ccf.org (A.P.F.); 3 Departments of Pathology, Cleveland Clinic, Cleveland, OH 44195, USA

**Keywords:** Graves’ disease, pretibial myxedema, mucin

## Abstract

A 64-year old male presented with a several-year history of an insidious-onset tender, itchy and xerotic rash on his lower legs. Past medical history was significant for Graves’ disease and Graves’ ophthalmopathy. The examination revealed peau d’orange-appearing plaques on his shins clinically consistent with pretibial myxedema. A punch biopsy showed separation of collagen bundles with extensive dermal mucin deposition, confirming the diagnosis of pretibial myxedema. After initially failing treatment with a topical clobetasol 0.05% ointment, the patient switched to regular pentoxifylline and triamcinolone 0.1% ointment under occlusion. He remains under follow-up.

## 1. Introduction

A 64-year old male presented to the dermatology department with a rash on both his lower legs. The rash had started several years before and was associated with tenderness, xerosis and itchiness. It had progressively worsened despite regular topical application of a triamcinolone acetonide 0.1% cream. On presentation, our patient denied any fever, weight loss, headache and other systemic symptoms. Past medical history was significant for Graves’ disease (diagnosed seven years prior to presentation), which was complicated by Graves’ ophthalmopathy. The patient had been treated with radioactive iodine, a total thyroidectomy and was taking maintenance-dose levothyroxine.

**Figure 1:**
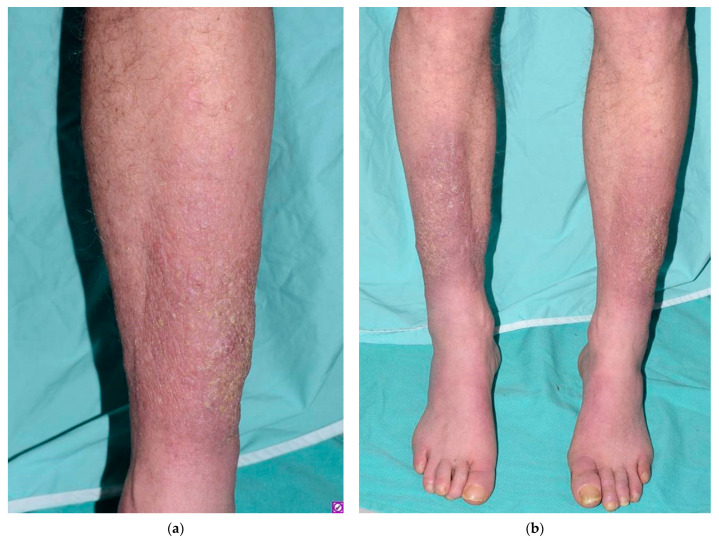
Indurated, pink, peau d’orange-appearing plaques and overlying adherent xerotic scale. (**a**) Closer view of the left shin; (**b**) both lower shins.

**Figure 2:**
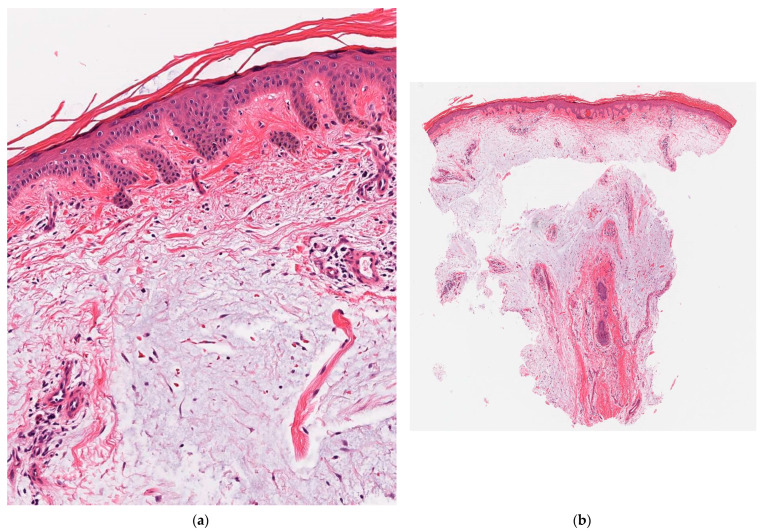
Hyperorthokeratosis overlying a mildly acanthotic epidermis with papillomatosis. There is significant separation of collagen bundles by large quantities of mucin with admixed fibroblasts within the dermis. (**a**) H&E ×2; (**b**) H&E ×15.

On physical examination, there were indurated, pink, peau d’orange-appearing plaques with an overlying xerotic scale on his shins (Figure 1a,b). Pedal pulses were palpable bilaterally. Other cutaneous manifestations of hyperthyroidism, such as thyroid acropachy, palmoplantar hyperhidrosis, alopecia and skin flushing, were not present. Clinically, the plaques were consistent with pretibial myxedema. A 4-mm punch biopsy showed separation of collagen bundles with extensive dermal mucin deposition with scattered fibroblasts (Figure 2a,b), confirming the diagnosis.

The patient was initially prescribed a clobetasol 0.05% ointment for once nightly application under occlusion on weekdays only, with a break on the weekends. After six weeks, the patient reported minor improvement in pruritus, although there was no appreciable improvement in appearance. The patient started taking 400 mg pentoxifylline tablets three times a day and switched to triamcinolone 0.1% ointment treatment consisting of a twice daily regimen under occlusion on weekdays only with a break every third week. The options of intravenous immunoglobulin (IVIG), rituximab and a trial of UVA-1 phototherapy were kept in reserve. He remains under follow-up.

## 2. Discussion

Pretibial myxedema affects 0.5–4.3% of patients with Graves’ disease [1], making it a relatively uncommon feature of the condition. The pathogenesis involves the thyroid-stimulating hormone (TSH) receptor autoantibody binding to and stimulating dermal fibroblasts that express the TSH receptor. The activated fibroblasts locally produce large quantities of glycosaminoglycans which draw fluid into the dermal space that ultimately leads to the expansion of the dermal connective tissue by a mucin-like substance [2].

Histological examination of pretibial myxedema classically demonstrates large amounts of mucin deposited within an edematous reticular dermis [3]. This is consistent with our case, which also showed scattered fibroblasts admixed within the mucin. Other differential diagnoses for localized pretibial lesions include focal cutaneous mucinosis (solitary), lymphedema, obesity-associated lymphedematous mucinosis and necrobiosis lipoidica. The main differentiating clinicopathological characteristics for pretibial myxedema are the association with Graves’ disease and the presence of extensive mucin deposition throughout the dermis extending into the subcutis without an inflammatory component (Table 1).

Additionally, the lack of lymphocytic infiltration in pretibial myxedema contrasts the extensive lymphocytic infiltration typically seen in the orbital adipose and muscle tissue of Graves’ ophthalmopathy [2]. However, features such as hyperorthokeratosis, acanthosis and papillomatosis are only occasionally seen in pretibial myxedema associated with Graves’ disease [4], making our patient a slightly unusual case of pretibial myxedema.

Treatment for pretibial myxedema is multiaxial, aimed at reducing risk factors (i.e., smoking) [5,6] and achieving euthyroid status [1]. The treatment of choice is topical application of a mid- or high-strength glucocorticoid under occlusion to improve absorption [7,8,9]. Pentoxifylline [10,11], intralesional injections of hyaluronidase with [12] or without [13,14] intralesional injections of glucocorticoid, rituximab [15], IVIG [16] and UVA-1 phototherapy [17] have all been reported with varying degrees of success.

## 3. Conclusions

In conclusion, we present a classic case of pretibial myxedema. Punch biopsy revealed extensive mucin deposition throughout the dermis separating collagen bundles with admixed scattered fibroblasts.

## Figures and Tables

**Table 1:** Characteristic clinical and histological features of localized pretibial lesions.

Lesion	Clinical Features	Histological Features
Pretibial myxedema	Seen in a minority of patients with Graves’ disease, more commonly affects females. Firm, non-pitting, scaly, thickened plaques or nodules are typically present on the shins or dorsum of feet. The skin may be slightly discolored and have a peau d’orange texture. Lesions can be pruritic and sore.	Extensive mucin deposition throughout the dermis and subcutis with collagen bundles widely separated or fragmented. Scattered dermal fibroblasts are often present without proliferation and there may be overlying epidermal hyperkeratosis and superficial perivascular lymphocytic infiltrate.
Focal cutaneous mucinosis (solitary)	Typically, an asymptomatic flesh-colored dome-shaped single papule smaller than 1 cm × 1 cm, most commonly found on the extremities. Most patients are between 29 and 60 years of age. It is slightly more common in males. In contrast, diffuse cutaneous mucinosis is associated with various systemic disease processes [18].	Characteristic focal mucin deposition in the upper dermis. Can extend into the deeper dermis but rarely into the subcutaneous fat. There may be an increase in scattered fibroblasts and capillaries within the lesion. The epidermis may be atrophic or hyperplastic and dermal dendrocytes are passively incorporated into the lesion [18].
Lymphedema	The primary disease results from malformation of lymphatic development and is rare. Secondary disease is acquired from damage to the lymphatic system. Parasitic filariasis is the most common global cause, although in developed countries, lymph node disruption, either surgical removal or irradiation, is the most common cause. There is a pitting edema in the affected limb with circumferential growth. Ulceration is not present, but there may be lymphatic vesicles and lymphorrhea. Skin can harden and thicken in the later stages [19].	Filarial infection shows keratinocyte hyperproliferation, focal acantholysis, lymphocytic infiltrate at the dermo–epidermal junction, dermal perivascular mononuclear infiltrate and subepidermal granulocytic infiltrates. There may be ‘lymphatic lakes’ between thick collagen bundles. Immunohistochemistry shows abundant macrophages (CD68+) and positivity for HLA-DR in all mononuclear and endothelial cells. Non-filarial lymphedema shows moderate keratinocyte proliferation, increased numbers of epidermal Langerhans cells (CD1+), moderate perivascular lymphocytic infiltrate and much less cellular positivity for HLA-DR [20].
Obesity-associated lymphedematous mucinosis	Can mimic pretibial myxedema but is classically associated with obesity; thyroid disorder is absent [21]. Typical skin-colored-to-yellowish papules, plaques and nodules arise in the pretibial region of lymphedematous legs [22].	Four distinct features have been identified: epidermal atrophy, moderate mucin deposition in the superficial dermis, vertically running angioplasia in the mid and superficial dermis and an increase in fibroblasts [21].
Necrobiosis lipoidica	Associated with type 1 diabetes mellitus, more commonly seen in females [23]. Characterized by enlarged firm red-brown papules that coalesce to form well-defined oval plaques with central yellowish-brown discoloration, atrophy and telangiectasias with a violaceous rim. Usually painless unless there is ulceration which may occur after trauma. Hypohidrosis and alopecia may develop within the plaque. Progression to squamous cell carcinoma has been reported [24].	Layered granulomatous inflammatory process alternating between zones of necrobiosis running parallel to the skin surface involving the full thickness of the dermis and extending into the subcutaneous fat septae. Collagen is degenerated and the epidermis is typically normal or atrophic. Necrobiotic areas are poorly defined with an intervening inflammatory infiltrate predominantly lymphocytic with plasma cells. No significant mucin deposition in the center of the granulomas [25].
